# Self-Reported and Actual Beta-Blocker Prescribing for Heart Failure Patients: Physician Predictors

**DOI:** 10.1371/journal.pone.0008522

**Published:** 2009-12-31

**Authors:** Sanjai Sinha, Mark D. Schwartz, Angie Qin, Joseph S. Ross

**Affiliations:** 1 Department of Medicine, James J. Peters VA Medical Center, Bronx, New York, United States of America; 2 Department of Medicine, Mount Sinai School of Medicine, New York, New York, United States of America; 3 Department of Medicine, VA New York Harbor Healthcare System, New York, New York, United States of America; 4 Department of Medicine, New York University School of Medicine, New York, New York, United States of America; 5 Department of Geriatrics and Adult Development, Mount Sinai School of Medicine, New York, New York, United States of America; Erasmus University Rotterdam, Netherlands

## Abstract

**Background:**

Beta-blockers reduce mortality among patients with systolic heart failure (HF), yet primary care provider prescription rates remain low.

**Objective:**

To examine the association between primary care physician characteristics and both self-reported and actual prescription of beta-blockers among patients with systolic HF.

**Design:**

Cross-sectional survey with supplementary retrospective chart review.

**Participants:**

Primary care providers at three New York City Veterans Affairs medical centers.

**Measurements:**

Main outcomes were: 1) self-reported prescribing of beta-blockers, and 2) actual prescribing of beta-blockers among HF patients. Physician HF practice patterns and confidence levels, as well as socio-demographic and clinical characteristics, were also assessed.

**Results:**

Sixty-nine of 101 physicians (68%) completed the survey examining self-reported prescribing of beta-blockers. Physicians who served as inpatient ward attendings self-reported significantly higher rates of beta-blocker prescribing among their HF patients when compared with physicians who did not attend (78% vs. 58%; p = 0.002), as did physicians who were very confident in managing HF patients when compared with physicians who were not (82% vs. 68%; p = 0.009). Fifty-one of these 69 surveyed physicians (74%) were successfully matched to 287 HF patients for whom beta-blocker prescribing data was available. Physicians with greater self-reported rates of prescribing beta-blockers were significantly more likely to actually prescribe beta-blockers (p = 0.02); however, no other physician characteristics were significantly associated with actual prescribing of beta-blockers among HF patients.

**Conclusions:**

Physician teaching responsibilities and confidence levels were associated with self-reported beta-blocker prescribing among their HF patients. Educational efforts focused on improving confidence levels in HF care and increasing exposure to teaching may improve beta-blocker presciption in HF patients managed in primary care.

## Introduction

Heart Failure (HF) was responsible for 3.3 million outpatient visits and approximately 300,000 deaths in the United States in 2006, with annual health care costs exceeding $37 billion. [1] A substantial body of randomized-controlled trial evidence has demonstrated that beta-blockers reduce mortality in patients with systolic HF. [2]–[5] Despite this trial evidence, and subsequent consensus clinical practice guidelines [6], many of these patients are not receiving beta-blockers [7], [8] and proven therapy has only slowly disseminated into practice. Understanding the gap between guidelines and prescribing patterns is crucial to delivering better care in this population.

Many factors may lead to underutilization of beta-blocker therapy for HF patients. For instance, patient insurance coverage, income, and medication co-payments impact the use of therapy [9], [10], although socioeconomic status was not associated with underutilization of heart failure treatment. [11] Other patient factors may also be associated with underutilization of beta-blocker therapy, such as advanced age, asthma, COPD, and depression. [12]–[15]

The impact of physician characteristics on guideline-appropriate care has been well studied for acute myocardial infarction [16]–[19] and asthma [20], [21], but has not been examined in the context of HF care, with the exception of physician specialty [8], [22], [23]. Physician demographics, including age [24] and years since residency, along with teaching responsibilities and confidence or self-efficacy [25], [26] may impact HF management. Physician training and sex were not associated with reported prescribing practice in asthmatics, but this has not been studied in HF patients. [27] Self-reported barriers to HF care by generalists (eg. confidence levels, co-management with cardiologists, fear of adverse reactions) were not correlated with prescribing angiotensin converting enzyme inhibitors (ACE-I) in HF, but may be related to prescribing beta-blockers. [28]–[30]

In order to examine the effect of physician characteristics on beta-blocker prescriptions, we used a cohort of heart failure patients seen at one of three New York City VA medical centers over a two-year period. Our aim was to examine the association between physician characteristics and their confidence in HF management, with self-reported and actual prescription of beta-blockers among patients with HF being managed within a primary care clinic.

## Methods

Ethics Statement: Regarding the physician survey, all participants were required to sign written informed consent prior to participating in the survey. This informed consent process was reviewed and approved by both the JJP VA Medical Center Institutional Review Board and the VA New York Harbor Healthcare System Institutional Review Board. We do not require a separate ethics statement.

We conducted a cross-sectional survey of primary care providers at three New York City VA medical centers, with supplementary retrospective chart review of a random sample of their HF patients. Written informed consent was obtained by all providers before their participation in the survey. The Institutional Review Boards at both the James J. Peters VA Medical Center and the VA New York Harbor Healthcare System (which includes both the NY and Brooklyn medical centers) approved of this study. In the United States, a physician assistant (PA) is a certified mid-level medical provider licensed to practice medicine with the direct or indirect supervision of a licensed doctor (an MD) or osteopathic physician (a DO). A 2-year masters degree is required to obtain the physician's assistant title. Similarly, Nurse Practitioners hold masters degrees and are licensed to practice with supervision. All health care providers (Physicians, Nurse Practitioners, and Physician Assistants) who were primary care providers for HF patients were eligible for inclusion. Because nearly all (98%) of these providers were physicians, we will refer to this group as physicians throughout this article. All physicians identified at the three medical centers were contacted via email and/or in person, and those providing informed consent were enrolled.

The questionnaire measured physician age, sex, VA site, specialty, and years since completing training. We asked physicians to self-report their HF practice patterns, such as use of beta-blockers and other medications for HF and confidence in HF management and referral rates to cardiology (see Appendix S1 for full survey).

The two main outcomes were: 1) self-reported prescribing of beta-blockers among HF patients, measured as the estimated proportion of HF patients in their clinical practice being treated with a beta-blocker; and 2) actual prescribing of beta-blockers among HF patients, dichotomized as currently versus not currently prescribed as documented in the progress note or pharmacy record for the prior visit closest to the survey date. For actual prescribing, we matched surveyed physicians to HF patients for whom they were identified as the primary care provider.

Data for this sample of HF patients were collected for a study that examined the association between beta-blocker use and patient characteristics, the details of which have been previously described. (12) Briefly, we trained two reviewers to use the VA's electronic medical record to abstract predefined patient information for all patients of surveyed physicians with International Classification of Disease, Ninth Revision (ICD-9) codes for systolic HF (428.x) seen in the primary care clinic between August 2002 and August 2004. Any visit to the patient's assigned primary care physician, including those not coded for HF, was counted toward inclusion in our sample. We excluded patients without a documented systolic ejection fraction (EF) less than or equal to 45% or who had fewer than 3 visits over the study period. Beta-blocker use in heart failure patients was a VA performance measure at the time of this study, but no electronic reminder was used to prompt prescribing.

For self-reported prescribing of beta-blockers among HF patients, we used non-parametric Spearman Correlation and Wilcoxon Rank-Sum tests to examine the independent effects of physician characteristics, including demographics, self-reported practice patterns, and confidence in HF management, on self-reported prescribing. For actual prescribing of beta-blockers among HF patients, we used Generalized Estimation Equation modeling to examine the independent effects of physician characteristics on actual prescribing, adjusting for patient characteristics our work had previously shown to be associated with beta-blocker use: patient age, ejection fraction, and past medical history of chronic obstructive lung disease, asthma, and depression. These analyses also accounted for the clustering (non-independence) of patients among primary care providers. All statistical tests were two-tailed, using a type I error rate of 0.05, and were performed using SAS 9.1 (SAS Institute, Inc., Cary, NC).

## Results

Sixty-nine of 101 physicians (68%) identified as practicing in the primary care clinics at one of the three VA medical centers completed the survey. Mean age was 45 years, 51% were female, 90% had a medical degree, and 81% had received clinical post-graduate training in primary care fields: family medicine or internal medicine without sub-specialty training, with the exception of geriatrics (Table 1). Nearly half (46%) were responsible for outpatient precepting (ie. Teaching) of post-graduate trainees at least once a week and 71% served as teaching attendings (ie. Supervising physician) on the inpatient hospital wards at least one month per year. On average, physicians reported that 23% of their patients had been diagnosed with HF; 30% were very confident in management of HF patients and 49% reported not often consulting cardiologists for management input for these patients. Mean self-reported prescribing rates among HF patients was 72% for beta-blockers, 84% for ACE-inhibitors, and 79% for diuretics.

**Table 1:** Physician Characteristics (n = 69).

Characteristic	No. (%)*
Mean age, y (IQ range)	45 (36–53)
Sex	
Female	35 (51)
Male	34 (49)
Clinical education	
Medical degree	62 (90)
Doctor of osteopathy or nursing degree	7 (10)
Clinical post-graduate training†	
Primary Care	51 (81)
Other Internal Medicine Subspecialty	12 (19)
Mean time since post-graduate training, y (IQ range)	13 (5–20)
Clinical practice site	
Bronx	31 (45)
Brooklyn	17 (25)
Manhattan	21 (30)
Teaching responsibilities include	
Outpatient precepting≥once per week	32 (46)
Inpatient wards ≥ one month per year	49 (71)
Mean estimated percentage of patients with HF, % (IQ range)	23 (10–30)
Frequency of consultation with a cardiologist when managing HF patients	
Not very often	34 (49)
Somewhat, moderately, or very often	35 (51)
Confidence in management of HF patients	
Not very, somewhat, or moderately	48 (70)
Very	21 (30)
Mean self-reported rates of prescribing among HF patients, % (IQ range)	
Beta-blockers	72 (60–90)
ACE-inhibitors	84 (80–95)
Diuretics	79 (70–95)

Note: IQ = Interquartile; HF = Heart Failure; ACE = Angiotensin-Converting Enzyme.

* Values expressed as number (percentage) unless otherwise noted. Percentages may not sum to 100 because of rounding.

† Among physicians with a medical degree or doctorate of osteopathy.

### Physician Characteristics and Self-Reported Prescribing of Beta-Blockers

Few physician clinical and demographic characteristics were significantly associated with self-reported prescribing of beta-blockers among HF patients, although significant associations for several other measures of confidence and experience in managing HF patients were observed (Table 2). Physicians whose teaching responsibilities included serving as attendings on the inpatient wards at least one month per year self-reported significantly higher rates of beta-blocker prescribing among their HF patients when compared with physicians who did not attend as often (78% vs. 58%; p = 0.002). In addition, we observed two associations that did not reach a significance level of p<0.05, as physicians whose teaching responsibilities included outpatient precepting at least once per week self-reported higher rates of beta-blocker prescribing when compared to physicians who did not precept as often (79% vs. 69%; p = 0.09) and physicians who had received a medical degree self-reported higher rates of beta-blocker prescribing when compared with physicians who had received a degree of osteopathy and nurses (74% vs. 54%; p = 0.07). There were no significant differences in self-reported rates of beta-blocker prescribing among HF patients for other physician clinical and demographic characteristics, including age, sex, clinical post-graduate training, years since post-graduate training, and site of clinical practice.

**Table 2:** Self-reported prescribing of beta-blockers among heart failure patients, stratified by physician characteristics (n = 69).

Physician Characteristics	Self-reported Rates, Mean (95% Confidence Interval)	P value
Age, y		0.21
30–39	78 (73–84)	
40–49	73 (64–82)	
50+	64 (51–77)	
Sex		0.15
Female	75 (68–82)	
Male	69 (61–76)	
Clinical education		0.07
Medical degree	74 (69–79)	
Doctor of osteopathy or nursing degree	54 (25–82)	
Clinical post-graduate training*		0.76
Primary Care	74 (68–79)	
Other Internal Medicine Subspecialty	73 (61–85)	
Years since post-graduate training		0.35
10 or less	74 (67–81)	
Greater than 10	69 (62–78)	
Clinical practice site		0.49
Bronx	71 (64–78)	
Brooklyn	70 (55–83)	
Manhattan	76 (67–85)	
Teaching responsibilities include		
Outpatient precepting ≥1 per week		0.09
No	69 (59–75)	
Yes	79 (73–83)	
Inpatient wards ≥1 month per year		0.002
No	58 (46–70)	
Yes	78 (73–82)	
Estimated percentage of patients with HF		0.07
Less than 20%	67 (58–75)	
20% or more	78 (72–83)	
Frequency of cardiology consultation when managing HF patients		0.07
Not very often	77 (71–83)	
Somewhat, moderately, or very often	67 (59–75)	
Confidence in management of HF patients		0.009
Not very, somewhat, or moderately	68 (61–74)	
Very	82 (76–88)	
Self-reported rates of prescribing ACE-inhibitors among HF patients		<0.001
Less than 90%	61 (52–70)	
90% or more	80 (75–85)	
Self-reported rates of prescribing diuretics among HF patients		0.005
Less than 90%	67 (60–74)	
90% or more	77 (70–84)	

Note: HF = Heart Failure; ACE = Angiotensin-Converting Enzyme.

*Among physicians with a medical degree or doctorate of osteopathy.

Physicians who were very confident in managing HF patients self-reported higher rates of beta-blocker prescribing among their HF patients when compared with physicians who were not (82% vs. 68%; p = 0.009). In addition, physicians who self-reported prescribing ACE-Inhibitors and diuretics among 90% or more of their HF patients self-reported higher rates of beta-blocker prescribing when compared with physicians who self-reported rates below 90% (ACE-Inhibitors: 80% vs. 61%; p<0.001; Diuretics: 77% vs. 67%; 0.005). Finally, we observed non-significant, but potentially important, associations in our two other measures of physician confidence and experience in managing HF patients, as physicians that estimated that 20% or more of their patients have been diagnosed with HF and physicians who consulted cardiologists least frequently for HF management self-reported higher rates of beta-blocker prescribing (p-values = 0.07).

### Physician Characteristics and Actual Prescribing of Beta-Blockers

Among 69 surveyed physicians, 51 (74%) were successfully matched to 287 of the 368 (78%) HF patients we had previously studied when examining associations between patient characteristics and beta-blocker use among HF patients.(12) Each physician was matched to an average of 5.6 patients (interquartile range: 2–8) for this analysis.

Of these 287 patients, 237 (83%) were currently prescribed a beta-blocker. There were no significant differences between physicians who were and who were not successfully matched to patients with respect to self-reported beta-blocker prescribing or any other clinical or demographic characteristic or measure of confidence or experience in managing HF patients with the exception of frequency of cardiology consultation, as 57% of physicians that were successfully matched to patients reported consulting cardiology not very often as opposed to 28% of physicians that were not matched (p = 0.03).

Few physician characteristics were significantly associated with actual prescribing of beta-blockers among HF patients (Table 3). Actual prescribing of beta-blockers was not significantly associated with physician age, clinical education, years since post-graduate training, practice site, or whether their teaching responsibilities included precepting in the outpatient clinics at least once per week or attending on the inpatient wards at least one month per year (p values>0.15), although there was a non-significant trend observed of female and non-primary care trained physicians being less likely to actually prescribe beta-blockers among their HF patients (p = 0.06 and p = 0.07, respectively). Similarly, actual prescribing of beta-blockers was not signficantly associated with physicians' estimated proportions of patients diagnosed with HF, frequency of cardiology consultation, or confidence in managing HF patients (p values>0.25). However, physicians with greater self-reported rates of prescribing beta-blockers among their HF patients were significantly more likely to actually prescribe beta-blockers to their HF patients (p = 0.02); a similar, non-significant relationship was observed for self-reported ACE-Inhibitor prescribing and actual beta-blocker prescribing (p = 0.07), but not for diuretic prescribing (p = 0.34).

**Table 3:** Regression estimates from independent Generalized Estimation Equation models examining the association between each physician characteristic and actual prescribing of beta-blockers (mean = 83%) among heart failure patients (n = 51 physicians and their 287 patients).*

Physician Characteristic	Odds Ratio	95% Confidence Interval	P value
Age	0.98	0.95, 1.01	0.25
Sex			
Male	ref	ref	
Female	0.50	0.25, 1.02	0.06
Clinical education			
Medical degree	ref	ref	
Doctor of osteopathy or nursing degree	2.39	0.60, 9.55	0.22
Clinical post-graduate training†			
Primary Care	ref	ref	
Other Internal Medicine Subspecialty	0.43	0.17, 1.08	0.07
Years since post-graduate training	0.97	0.94, 1.01	0.20
Clinical practice site			
Bronx	ref	ref	
Brooklyn	1.53	0.68, 3.45	0.31
Manhattan	1.45	0.63, 3.38	0.38
Precepts in outpatient setting ≥1 per week			
Yes	ref	ref	
No	0.61	0.30, 1.24	0.17
Inpatient wards ≥1 month per year			
Yes	ref	ref	
No	1.07	0.39, 2.92	0.89
Estimated percentage of patients with HF	0.99	0.97, 1.01	0.38
Frequency of cardiology consultation when managing HF patients			
Not very often	ref	ref	
Somewhat, moderately, or very often	0.99	0.49, 2.00	0.97
Confidence in management of HF patients			
Not very, somewhat, or moderately	0.69	0.36, 1.34	0.28
Very	ref	ref	
Among HF patients, self-reported rates of prescribing			
Beta-blockers	1.02	1.00, 1.04	0.02
ACE-inhibitors	1.03	1.00, 1.05	0.07
Diuretics	1.01	0.99, 1.02	0.34

Note: HF = Heart Failure; ACE = Angiotensin-Converting Enzyme.

* Generalized Estimation Equation models were additionally adjusted for patient age, ejection fraction, and past medical history of chronic obstructive lung disease, asthma, and depression.

† Among physicians with a medical degree or doctorate of osteopathy.

## Discussion

Improving physician guideline adherence in HF care is an important performance measure within the VA health care system as well as nationally. Our current research focused on identifying physician characteristics as predictors of both self-reported and actual beta-blocker prescription. We found that primary care physicians reported high rates of beta blocker prescribing (72%) in their HF patients and had even higher rates of actual prescription (83%). Although a number of individual physician characteristics were associated with greater self-reported rates of beta-blocker prescribing to HF patients, none of these characteristics were associated with greater actual rates of prescribing. The only characteristic that was associated with greater actual prescribing rates was greater self-reported prescribing rates. We cannot be certain that self-reported prescribing is reflecting physician's self-perception of their practice patterns or ideal practice, but the similar rates of self-reported and actual prescribing suggest it represents self-perception. Self-reported prescribing remains an interesting outcome to study for two reasons. First, it is a measure of physicians' understanding of their own prescribing behavior. Second, it may also be a proxy of physician practice patterns, conveying some amount of information of how physicians practice “in general”, as opposed to for each individual case.

Understanding which physician characteristics are associated with greater self-reported prescribing of beta-blockers for patients with HF can inform future efforts to improve care. We found that physicians who were more confident in their own skills at managing patients with HF had higher self-reported prescribing rates, suggesting that interventions to increase confidence, such as education programs, may lead to improved care. This finding was additionally reflected by a non-significant trend we observed of greater self-reported prescribing rates among physicians who consulted specialists less often for HF care. Similarly, we found that physicians with teaching responsibilities also had higher self-reported prescribing rates, reinforcing the common wisdom that to teach is to learn. Our findings are also consistent with prior research that has shown increased confidence is associated with greater self-reported prescribing rates of ACEIs among heart failure patients. [30] Quality improvement programs should consider focusing on increasing confidence in HF management amongst primary care physicians, perhaps through CME activities, lecture series, journal clubs, or other HF- based learning modules on a regular basis.

We had a unique opportunity to explore actual beta-blocker prescribing rates within a sub-sample of our physicians to test whether the same characteristics that were associated with self-reported prescribing rates were also associated with actual prescribing rates. None of the characteristics we examined, including those characteristics that predicted self-reported prescribing such as confidence and teaching responsibilities, were associated with actual prescribing rates, with the exception of self-reported rates. These findings are somewhat perplexing but our study was limited by a small sample size and possibly did not have the power to detect true differences in actual prescribing rates between physician subgroups. However, our analyses are exploratory and inform future research.

There are other considerations in interpreting our results. First, we only examined beta-blocker prescribing for patients with HF and did not address the impact of physician characteristics on other important dimensions of HF care quality, such as use of other recommended medications or non-pharmacological treatments, patient experiences and satisfaction with care, or patient outcomes. Second, we examined self-reported and actual prescribing rates, but were unable to examine actual patient adherence to prescribed medications. Physician characteristics may also be associated with patient adherence to prescribed therapy. Finally, our study was limited to physicians and patients at three New York City-based VA medical centers and may not be generalizable to the general population. Importantly, all VA medical centers use an integrated electronic medical record system that includes physician reminders to prompt use of recommended preventive and chronic disease care, although no prompts address beta-blocker prescription among outpatients with HF. Nevertheless, rates of beta-blocker prescribing may be higher than in the general population.

Our findings suggest that some physician clinical characteristics such as teaching responsibilities and confidence levels, are associated with self-reported beta-blocker prescribing in HF patients. Targeting these characteristics by improving education of primary physicians, and increasing their exposure to teaching may improve beta-blocker presciption in HF patients followed in primary care settings.

## Supporting Information

Appendix S1 Provider Survey
(0.03 MB DOC)
