# Supplementary material for: Self-Reported and Actual Beta-Blocker Prescribing for Heart Failure Patients: Physician Predictors
Source: PLoS One. 2009 Dec 31;4(12):e8522. doi: 10.1371/journal.pone.0008522 (PMC2796176; doi:10.1371/journal.pone.0008522)
Supplement: Appendix S1 — Provider Survey (0.03 MB DOC) [file pone.0008522.s001.doc]

**Appendix S1**

# PROVIDER SURVEY

# “BUSH: Beta-blocker Utilization in Systolic Heart Failure”

Thank you for agreeing to complete this brief survey. Your participation will help us understand how to better treat our patients with Congestive Heart Failure (CHF). Your identification will be kept confidential, and your responses will be used only for research purposes. **Please don’t forget to sign the last page of the informed consent by the ‘X’.** Thanks again!

1. Approximately what percentage (0-100) of your patients do you estimate have chronic/congestive heart failure(CHF)? _______%

*The next set of questions will deal with comorbidities that might affect your decision in using beta-blockers in CHF.*

2. What proportion of your patients with CHF and **COPD** would you place on a beta-blocker:

1 2 3 4 5

0-20% 21-40% 41-60% 61-80% 81-100%

3. What proportion of your patients with CHF and **asthma** would you place on a beta-blocker:

1 2 3 4 5

0-20% 21-40% 41-60% 61-80% 81-100%

4. What proportion of your patients with CHF and **peripheral vascular disease** would you place on a beta-blocker:

1 2 3 4 5

0-20% 21-40% 41-60% 61-80% 81-100%

5. What proportion of your patients with CHF and **depression** would you place on a beta-blocker:

1 2 3 4 5

0-20% 21-40% 41-60% 61-80% 81-100%

6. What proportion of your patients with CHF and **fatigue** would you place on a beta-blocker:

1 2 3 4 5

0-20% 21-40% 41-60% 61-80% 81-100%

7. What proportion of your patients with CHF and **diabetes** would you place on a beta-blocker:

1 2 3 4 5

0-20% 21-40% 41-60% 61-80% 81-100%

8. What proportion of your patients with CHF and **erectile dysfunction** would you place on a beta-blocker:

1 2 3 4 5

0-20% 21-40% 41-60% 61-80% 81-100%

9. What proportion of your patients with CHF and **dizziness or syncope reported in the past 6 months** would you place on a beta-blocker:

1 2 3 4 5

0-20% 21-40% 41-60% 61-80% 81-100%

10. What proportion of your patients with CHF and **asymptomatic bradycardia** would you place on a beta-blocker:

1 2 3 4 5

0-20% 21-40% 41-60% 61-80% 81-100%

11. What proportion of your patients with CHF and **hypotension reported in the past 6 months** would you place on a beta-blocker:

1 2 3 4 5

0-20% 21-40% 41-60% 61-80% 81-100%

*Next, are some general questions about your approach to managing CHF patients.*

12. How often do you refer to, or consult with, a cardiologist when managing CHF patients?

1 2 3 4

not very somewhat moderately very

often often often often

13. How *confident* are you that you are up to date regarding management of CHF?

1 2 3 4

not very somewhat moderately very

confident confident confident confident

14. What percentage (0-100) of your patients with CHF would you estimate are currently on…?

14a. an ACE Inhibitor? ___________%

14b. a Beta-blocker? ______________%

14c. a diuretic? __________________%

15. Do you precept residents on a weekly basis in an outpatient clinic? 1=Yes 2=No

16. Did you attend on the wards *at least one month* over the past year? 1=Yes 2=No

*Finally, some questions about you.*

17. Your name: ­­­­­_____________________________________________

18. Your gender: 1=Male 2=Female

19. Your age: __________

20. Which V.A. do you work at? 1=Bronx 2=Brooklyn 3=Manhattan

21. Type of Provider:

1=MD 2=DO 3=NP/PA

21. If MD or DO, what is your practice specialty?

1= Internal Medicine 2=Cardiology 3=Other ________________ 4=N/A

22. If MD or DO, number of *years* since you completed your residency training: __
